# Specific Interventions for Implementing a Patient-Centered Approach to TB Care in Low-Incidence Cities

**DOI:** 10.3389/fmed.2019.00273

**Published:** 2019-11-26

**Authors:** Adrià Pujol-Cruells, Cristina Vilaplana

**Affiliations:** ^1^Experimental Tuberculosis Unit, Fundació Institut Germans Trias i Pujol, Universitat Autònoma de Barcelona, Badalona, Spain; ^2^Centro de Investigación Biomédica en Red de Enfermedades Respiratorias (CIBERES), Madrid, Spain

**Keywords:** tuberculosis, patient-centered approach, care management, interventions, socio-economic factors, anthropology

## Abstract

**Background:** According to the latest Guidelines from the World Health Organization, there is an increasing need for patient-centered tuberculosis disease management given the socio-economic factors influencing the tuberculosis epidemic. In the present study, we aimed to study TB in Barcelona city from an anthropological point of view and to devise a series of specific proposals to implement a patient-centered approach in our setting.

**Methods:** We carried out a qualitative study using an anthropological approach in Barcelona in the period between November 2017 and November 2018 and proposed specific interventions based on our observations.

**Results:** In practice, in our environment (a low-incidence European country where tuberculosis tends to present in patients with multiple social problems), and despite the goodwill of the care teams, there are no established and stable circuits, or specific tools to ensure that this is done routinely. Based on our observations, we have devised a series of specific proposals to implement a patient-centered approach. With these interventions we aim to (a) directly ameliorate TB patients well-being in any diagnostic/healthcare management center and (b) at more general level, to increase TB detection and treatment adherence.

**Conclusions:** The patient-centered TB management recommended by the WHO might be essential for patients' well-being, but there is a lack of circuits or working protocols that ensure its implementation in a regulated manner. In the present manuscript we explain the various concrete measures that we propose in our region and which could be put into practice in other cities or geographic regions with similar epidemiological characteristics.

## Introduction

According to the latest global report on tuberculosis (TB) published by the World Health Organization (WHO), there were up to 10 million cases of TB and 1.3 million TB-related deaths in 2017 (1). Following the global trend, the incidence rate in Europe is decreasing. In Spain, after two epidemic peaks, coinciding first with the abundance of parenteral drug users among people infected with HIV (in the late 1980s and 1990s), and then with the large-scale migration from countries with a high incidence of TB found in the early 2000s, TB is relatively well-controlled and is also decreasing in incidence (2). As such, the main problems in epidemiological terms are the high incidence in large cities and delays in diagnosis (an average of 60 days from the onset of symptoms according to the latest data available) (3). In the case of Barcelona, a model TB control program established by the city's Public Health Agency, which allows an interchange of information between clinical TB units, case managers and public health nurses, as well as healthcare agents (4), thereby facilitating the active monitoring, detection and follow-up of patients and access to the complete data for all cases, has been operating for more than 30 years. This has led Barcelona to be considered a laboratory in which the problems and challenges of TB control programs in Western Europe, especially in large cities, can be studied. In the latest report (with data from 2016), the incidence rate was 16.2/100,000 inhabitants, distributed unevenly in the various districts depending on their levels of income (up to 43.8/100,000 in the Ciutat Vella district, for example). About half of these patients were born outside Spain, the percentage of patients with socio-economic vulnerability was estimated at 7.3% and there was an increase in homelessness and alcoholism. The percentage of patients with completed treatment was lower in the elderly, the homeless, those addicted to parenteral drugs and those with a history of imprisonment, partly due to the high mortality in these groups (3).

One of the aspects that the latest WHO report highlighted is that, in order to achieve the reductions in incidence necessary to meet the milestones set for 2030, the socio-economic factors influencing the TB epidemic must be addressed (1). Unfortunately, this is not the only problem, as the latest WHO publication regarding TB treatment guidelines (5) clearly suggests that a patient-centered approach is required in the treatment of TB patients. Such an approach considers that it is insufficient to cure only the illness and that the patient must also be cured. For this: (1) the patient must participate actively in the process, (2) their preferences, needs and values must be taken into account, and (3) a complete approach, including both mental health and social aspects, must be used. There is sufficient evidence to support this more holistic-type approach in TB has been shown to have an important impact on both patient well-being and treatment outcomes (6).

In the present manuscript we aimed to study TB in Barcelona city from an anthropological point of view and to devise a series of specific proposals to implement a patient-centered approach; applicable to our setting but which could be extrapolated to other cities with similar epidemiological situations.

## Methods

We carried out a qualitative study using an anthropological approach in Barcelona in the period between November 2017 and November 2018. The field work was divided into three phases. First phase was floating observation of 40 patients and 8 health professionals: initial phase of prospecting, without intervention. The second phase was participatory observation: real follow-up including informal conversations and unstructured interviews with patients diagnosed with TB (*n* = 22), and health professionals including medical and nursing staff, social workers and health agents (*n* = 29). No data from the participants was recorded, only the anthropologist impressions. The third phase was collaborative observation: proposing strategies for intervention in the community.

The study was conducted within the STAGE-TB project (registered at ClinicalTrials.gov, identifier: NCT03691883), coordinated by the Experimental Tuberculosis Unit; and its protocol and procedures were reviewed and approved by the correspondent responsible institutional review committees.

## Results

Our qualitative study confirmed the high prevalence of social exclusion associated with this disease. In Barcelona, the paradigmatic patient is an immigrant with difficulties entering the social and working environment of the region and with language-related communication problems. The social exclusion associated with TB is determined by both the personal, family and legal status of the individual (whether native or migrant) as well as by the added problem of the disease itself, which given the resulting chronicity, associated disability and hospitalization (when required) acts as a barrier (not always symbolic) that hinders or prevents the patient from finding work, having a place to live and establishing social relationships. Healthcare workers recognize well-situations of poverty, malnutrition, social exclusion, and fragility at a mental health level; but there are no established circuits or protocols that allow addressing appropriately these situations.

Moreover, hospitalization or the termination of any activity considered normal for the individual causes the patient to feel trapped in limbo, which can hinder their reintegration into the environment, especially the working environment. In addition, there are two cultural perceptions around the disease: in stabilized environments, TB is associated with pathological social states and is therefore seen as an anomaly, whereas in less structured social environments, TB is perceived as a consequence of the pervading conditions. The social exclusion (and socio-economic factors in general) associated with the illness, as well as the duration of medical treatments, also has an impact on the institutions and health personnel that these patients attend, and they are often not aware of the quality of the service they receive.

Depending on the different geographical origins of the patients, there is also a cultural shock between the patients and their new social framework (which also includes the area of healthcare), since the disease brings individuals into contact with cultural realities to which they are not accustomed, mainly due to differences in customs and gender perspectives. This generates isolation in these patients and means that, although they tend to recognize the seriousness of the illness (and its associated medical problems), this perception does not always translate into healthy behaviors. In order to minimize this cultural shock and its impact on individuals, the design of activities in which patients can participate is often necessary. However, the offer is limited and must take into account the cultural point of view.

Based on our observations, we have devised a series of specific proposals to implement a patient-centered approach, applicable to Barcelona and which could be extrapolated to other cities with similar epidemiological situations. With these interventions we aim to a) directly ameliorate TB patients well-being in any diagnostic/healthcare management center and b) at more general level, to increase TB detection and treatment adherence. These proposals are set out in Table 1 and can be divided depending on the level at which they are to be applied: (a) at the diagnostic and/or treatment center, which can improve the conditions of the patients directly, and (b) at the population level in general, affecting neighborhoods with the highest risk of TB.

**Table 1:** Specific interventions proposed to implement the patient-centered approach to TB care.

**Level of intervention**	**Proposals**	**Expected impacts**
In diagnostic or treatment centers	Scheduled activities (leisure, health, training, psychological support) available to TB patients, adapted from a cultural point of view (gender, age, religion, customs, etc.), performed in suitable spaces, in-house or in neighborhood, or city facilities (libraries, museums, swimming pools, gyms) thanks to collaboration agreements.	For patients: To increase patient well-being and mental balance, favor their integration into society, establish a bond with the city, minimize culture shock, decrease para/illegal behaviors. For society in general: increase awareness. For centers: to offer a more complete portfolio covering the patient-centered approach.
	Regularly scheduled institutional group sessions with both patients and staff attending.	Two-way flow of information; to reveal problems, doubts and fears; increase treatment adherence.
	Social mentoring activities, in-house or outsourced, thanks to collaboration agreements with other institutions or NGOs.	To accelerate and increase reinsertion, emancipation and adherence to treatment, while reducing isolation and unhealthy habits, in the framework of interventions to generate, reconnect or strengthen the bonds of the individual with society.
In high TB incidence neighborhoods	Information tools (i.e., leaflets, infographics, videos), in different languages, of universal access, patients coauthoring. Two versions: one for patients and their relatives, another one for the general population.	For patients: to increase treatment adherence, bond with healthcare staff and increase patient confidence. For relatives: to resolve doubts and give them tools to handle day-to-day problems. For the general population: to increase awareness, to promote early diagnosis.
	Conferences, seminars, informal talks on the disease, its management and its impact; patients involved; conducted using community infrastructures and spaces: schools, civic centers, libraries, museums, places of worship, leisure and health facilities (gymnasia, swimming pools, shopping centers).	To increase awareness of the presence of the disease in the environment; increase health literacy; reduce fears and resolve doubts.
	Continuing education for healthcare professionals on TB screening, diagnosis, management and treatment; carried out periodically; i.e., refresher courses, infographics, etc.	To increase awareness of the presence of the disease and reduce diagnostic delay; to refresh and update information on TB management.
	Creating interdisciplinary teams; at city, regional or national level; recommended to include healthcare workers, mental health therapists, social science workers, nutritionists, and patients (expert patient).	To generate documents, protocol circuits, and guidelines that ensure the implementation of a global approach to the management of TB patients.

**Figure 1:**
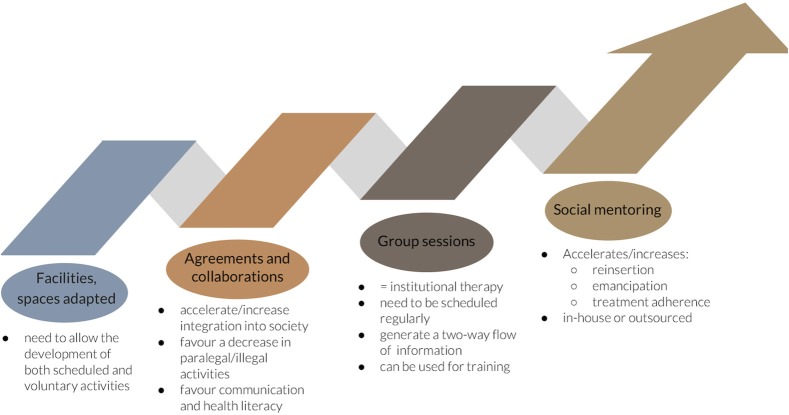
Interventions proposed at the level of the diagnostic and/or treatment center.

At the level of the diagnostic and/or treatment center (Table 1 and Figure 1), it is essential to carry out activities that mitigate unemployment or the illegal behavior, stigma and uprooting of patients, especially if they are admitted. These activities must include leisure activities, health, training, and psychological support (programmed; compulsory or voluntary) and be adapted to the patients' cultural context (gender, age, religion, customs, etc.). Moreover, the centers must be equipped with suitable facilities and spaces to carry them out and there must be a network of agreements and collaborations between the centers and the facilities in the city, and particularly in the patients' neighborhood. At an internal level, we believe that both the presence of a regularly scheduled institutional therapy in the form of group sessions attended by both patients and healthcare staff and social mentoring program (in-house or outsourced through contracts or collaborations with mentoring associations) should be implemented.

**Figure 2:**
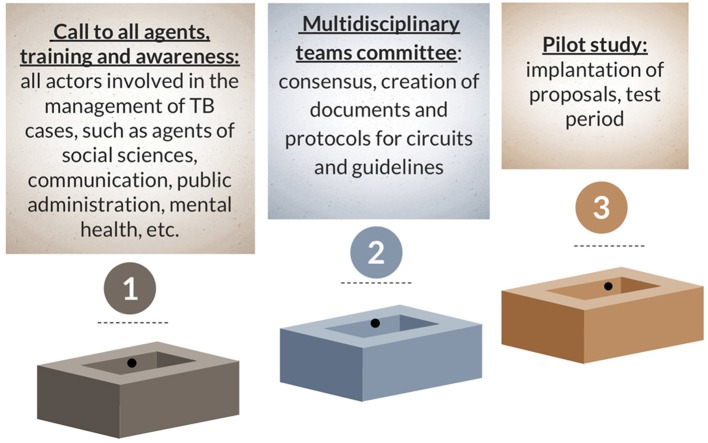
Creation of interdisciplinary teams: phases.

With regard to the interventions of a more general nature proposed for application in neighborhoods with the highest incidence of TB (Table 1), it would be important to have information tools (i.e., leaflets, infographics, videos) in different languages (in Barcelona: in Catalan, Spanish, French, English, Arabic, Urdu, Chinese) and universal access, with the participation of patients during their design; to organize talks and conferences on the disease in which patients or ex-patients also participate, thereby taking full advantage of the figure of the “expert patient”; and to ensure the continuing specialized training of professionals. Given the profile of an individual potentially affected with TB (with the concomitant social pathology that affects detection, diagnosis and treatment adherence), we believe that the creation of interdisciplinary teams from professionals already working on the ground would be essential (Figure 2).

The holistic and multidisciplinary approach proposed requires substantial human and financial resources. Unfortunately, a concrete estimation of its cost is very difficult to be calculated, as it depends on the setting where to be implemented and the resources available (including the type of Healthcare System). However, we made the exercise of detailing the items which in our opinion should be considered when budgeting the different proposed interventions. We have included this information as Table 2, which could be used to calculate the amount of funding needed once applied to the cost of living, cost of salaries, and other cost measures of the specific country/region where to implement the intervention.

**Table 2:** Suggested items to be considered when budgeting the specific interventions proposed.

**Interventions proposed**	**Items to be considered when budgeting**
Scheduled activities available to TB patients	If done in-house: costs of adapting/transforming the in-house spaces to make them suitable for the purposes. salary of professionals organizing and/or conducting the activities or effort cost. Costs can be diminished/suppressed by establishing collaboration agreements with neighborhood or city facilities (libraries, museums, swimming pools, gyms).
Regularly scheduled institutional group sessions with both patients and staff attending.	Salary for a psychologist/therapist.
Social mentoring activities, in-house or outsourced, thanks to collaboration agreements with other institutions or NGOs.	Salary for a program supervisor (administrative management of the team, formative supervision, monitoring performance). Salary for mentors. Mentors' travel expenses (including per diem). Communication support (i.e., Mobile phones, internet connection). Cost of training the mentors. These costs could be decreased/ suppressed by establishing collaboration agreements with local associations and NGO to partially cover the activity.
Information tools for patients and relatives and for the general population.	Costs of designing the information material (payment-as-a-service or effort cost of professionals). Costs of translations. Costs of publishing the material. Costs of distributing the material (salary or effort cost of distributor/s).
Communication activities	Effort cost of professionals giving the communication activities. This cost can be diminished/suppressed if the professionals agree to voluntary work. Cost of renting community infrastructures and spaces. This cost can be diminished/suppressed by establishing collaboration agreements with city council or similar local institutional bodies.
Continuing education for healthcare professionals on TB	Costs of performing an educational course. It will depend on the format and channel: webinar, videos, tutorials, classroom course. Can include effort cost of professionals involved (including professors), internet domain, editing. Costs can be diminished by establishing collaboration agreements with universities, foundations and NGO which can contribute to the activity. Costs of designing educational material (payment-as-a-service or effort cost of professionals) and publishing educational material.
Creating interdisciplinary teams	Minimal team: Effort cost of 1 MD to coordinate the program and to act as the interlocutor with the physicians of hospitals and other institutions feeding the program with patients. Effort cost for 1 nurse to act as the interlocutor with the MD in charge of the program and the nurse team at territorial level about the specific cases. Salary of 1 healthcare manager/administrative to manage the team and to coordinate the collaboration agreements with the institutions and facilities. Effort cost for 1 healthcare worker. Effort cost for 1 social assistant. Effort cost for 1 expert patient and/or costs of meetings with expert patients. Minimal team needs coordination with mental health therapists and nutritionists, that in case of being included in the team their effort cost or salary would need to be also included.

## Discussion

One of the limitations of the study is that we focused on the anthropological approach and didn't conduct an associated epidemiological study to perform a precise description of the studied population (including their geographical origin, spoken language and detailed social condition). However, our observations were in line with results of the quantitative studies carried out in this city (2, 3, 7) and similar to findings from other major European cities (8, 9). The treatment management options recommended by the WHO in its guidelines are currently applied well in Spain [in Barcelona, for example, the percentage of cured bacillary pulmonary cases or those who have completed treatment is as high as 92.5% (3)], except for the administration of DOTS (performed in 30.4% of patients, reaching 71% in users of injected drugs (UDI) and 90% in homeless people, although rarely via telematics) and the lack of uniformity in terms of supply.

The main problem arises, however, when it comes to treatment-adherence interventions. The WHO recommendations (5) regarding the provision of health education and advice to patients are met relatively well in Spain by all persons involved (patients and healthcare personnel). However, there are no guidelines or recommendations for activities to ensure the uniformity of actions and their fulfillment. This situation worsens when it comes to recognizing and addressing situations of poverty, malnutrition, social exclusion and fragility at a mental health level. Thus, although these problems are usually correctly identified at a healthcare level, it is often logistically difficult to resolve them and, if this is achieved, it is often solely due to the goodwill of all those involved (medical and nursing staff, health agents, social workers at the centers and associations). Moreover, there is currently little or no coordination or uniformity, and there are no established circuits or protocols that allow comprehensive care to be provided. Thus, although there are links to other public-health programs and sufficient resources to properly address patients with co-morbidities (such as HIV co-infection), other problems, such as those from the mental health sphere, are often under-diagnosed and, therefore, under-treated.

Another of the main problems of TB in our region is that, despite being aware of the precarious socio-economic factors present in some patients, it is difficult to include these patients, as requested by the WHO, in publicly offered services and community-based resources and support, mainly due to the lack of established protocols and circuits.

Given this situation at the local level of a large city (Barcelona), as seen from our anthropological work, and taking into account the recommendations of the WHO (5) and those of “The working group for TB control in big cities and urban risk groups in the EU” (9), we have devised a series of specific proposals applicable to Barcelona that could be extrapolated to other cities with similar epidemiological situations.

Interventions proposed at the level of the diagnostic and/or treatment center, apart from facilitating the leisure options and training available at the centers, and working on how to stop transmission and spread of the disease, ensure that there is no discontinuity between the hospital/residential world and that of the general population outside this institutional context. It also adds value to those centers that offer it, since the quality of the services provided increases beyond strictly medical care (which is already covered nowadays). At an internal level, regularly scheduled institutional therapy help strengthen messages related to the disease, encourage a two-way flow of information and can be used to carry out training. They also have an important impact on the outcomes of the disease (10). This type of activity could form part of the group of interventions that favor treatment adherence and the psychological support defined by the WHO (5). Finally, we propose that a social mentoring program should be implemented, to accelerate and increase reintegration, emancipation and treatment adherence, while reducing both isolation and unhealthy habits, in the framework of interventions to generate, reconnect or strengthen the ties of individuals with society (11).

With regard to the interventions of a more general nature proposed for application in neighborhoods with the highest incidence of TB, we must make an effort to intensify the presence and implementation of tools that enhance visibility of the disease given the fact that poor population information and inadequate training of professionals result in a delay in diagnosis. In this sense, it would be important to ensure the participation of patients during their design. Similar experiences have shown that joint development (by professionals and patients working together) of the information (the story) plays a key role in the solution to the problem (12, 13).

All these communication tools and activities should applied twice: once for patients and their friends and families, and the second time for the general population (distributed in geographic regions at higher risk). In this regard, it should be noted that, in order to optimize resources, it would be advisable to use existing resources that have proven useful in other regions or countries with a similar TB incidence and characteristics. All these initiatives need to be carried out using community infrastructures and space in order to link them to society itself, especially schools, civic centers, libraries, places of worship, and leisure and health facilities (gymnasia, municipal swimming pools, shopping centers). The continuing specialized training of professionals is also indispensable (5), especially in low-impact countries (such as those in Western Europe), and can be done by way of refresher courses or much simpler tools to encourage professionals to take into account the illness in these environments, such as infographics and reminders in the clinic.

On the creation of interdisciplinary teams from professionals already working on the ground, we do believe it would represent a significant step toward tackling the illness and increasing patient wellbeing. These teams could be created at a city, regional or national level, and it may be advisable for them to be led by local Public Health Agencies. Although this approach has not been tested in tuberculosis in our setting, similar initiatives, such as the management of care for people with complex needs, which involves the coordination of health services (including mental health) at both the primary care and hospital levels, social services (both at a community and an individual level), and a specialized community support team (formed by geriatricians, pharmacists, occupational therapists, dieticians), have been implemented (14).

To accomplish this goal, the following sequence of steps would be required: (1) a call to all agents, including the main actors involved in TB care (professionals in the fields of social sciences, public administration, politics, and communication, from the legislative world, criminologists, teachers from all walks of life (especially adults), nutritionists, therapists and others). In this first session, all these agents would be summoned and trained, and they would be invited to put forward their reflections and proposals at a second meeting. A Multidisciplinary Teams Committee, in other words a group of people from all the different levels that would lead the initiative and ensure its good performance, would also be created. The second step would be the pooling of new approaches and points of view from all these other disciplines in order to create a pilot team that can work on the search for improvements and solutions for patients, reaching a consensus and generating documents, along with protocol circuits and guidelines that ensure a global approach to the disease. Again, we emphasize that both the screening for mental disorders and counseling and psychological support for patients are very important for the entire duration of disease follow-up and treatment, especially in those most vulnerable at the social level [such as refugees or immigrants (15) or in cases of multi-drug resistance (16, 17)], and these need to be both offered and provided, then routinely scheduled in a regulated manner.

Finally, under the tutelage of the Multidisciplinary Teams Committee, and once all proposals have been analyzed, the interdisciplinary pilot team should be appointed, its ideal test performance and main lines of work (areas, agents) defined, and the proposals implemented in the territory during a trial period. Although initially there may be multiple solutions for financing these teams (including the association of this initiative with a research project), it is important that the local public administration assumes responsibility for the economic viability of these teams beyond the pilot stage.

From an economic point of view, we have envisaged several interventions with a potential wide range of feasibility depending on the available resources at local or governmental level. As the funds for the implementation of actions might be unassumable by the diagnostic/healthcare management centers and the National Health System (which can vary a lot between different countries sharing the same epidemiology in terms of TB), collaboration agreements with local associations, NGO, city council or similar local institutional bodies need to be established, in order to partially cover or contribute to the activity, and thus to decrease or even suppress part of the costs. Further studies should be conducted in order to elucidate at which extent some of the actions proposed here could also be implemented in low-income countries, which due to their high-TB prevalence could benefit even more of their impact if proven successful.

## Conclusion

The WHO recommendations in terms of tackling TB are very meaningful from the point of view of addressing the suffering of patients beyond the disease itself, and although care teams are usually aware of this, there are currently still no circuits or working protocols that define the necessary interventions or circuits that ensure their implementation in a regulated manner. With this publication, we hope to highlight this shortcoming while explaining the various concrete measures that we propose in our region and which could be put into practice in other cities or geographic regions with similar epidemiological characteristics, once adapted to the limitations of the new setting.

## Take Home Message

There is an increasing need for patient-centered tuberculosis disease management, but this is not done routinely. In this article we propose a series of specific interventions that could be easily implemented in low-incidence cities during care management.

## Data Availability Statement

All datasets generated for this study are included in the article/supplementary material.

## Ethics Statement

The studies involving human participants were reviewed and approved by the correspondent responsible institutional review committees, both the Germans Trias i Pujol Hospital Ethics Committee (study approval code: PI-17-064) and the Vall d'Hebron Hospital Clinical Research Ethics Committee (code PR(AG)101/2017). Verbal informed consent was obtained from all participants because the study was based on informal conversations, no questionnaires, or structured interviews were conducted and no personal data from the participants were recorded. Written informed consent for participation was not required for this study in accordance with the national legislation and the institutional requirements.

## Author's Note

We would like to emphasize that all the reflections presented have been possible thanks to TB patients, all healthcare staff involved, Servicios Clínicos SLU, the Barcelona Public Health Agency, and the health agents of Barcelona. Preliminary results of the study were presented at the XXII International Workshop on TB conducted in Barcelona on the 26–27 November 2018, and published as a conference abstract in Rev Enf Emerg 2018;17:159–160.

## Author Contributions

AP-C conducted the floating and participatory observation phases of the field work. Both AP-C and CV contributed in the design of the study, worked in the analysis and interpretation together, and in the collaborative observation by proposing strategies for intervention in the community. The authors worked together to draft the manuscript and approved its final version.

### Conflict of Interest

The authors declare that the research was conducted in the absence of any commercial or financial relationships that could be construed as a potential conflict of interest.
